# Role-Differentiated AI Competencies and Curriculum Implications for Health Professions Education: Qualitative Study

**DOI:** 10.2196/97608

**Published:** 2026-09-08

**Authors:** Yuyi Park, Yejin Han, Hyeongjo Kim, Solmoe Ahn, Frederick William Kron, Jihyun Lee

**Affiliations:** 1 Department of Dental Education, School of Dentistry and Dental Research Institute, Seoul National University, 103 Daehak-ro, Jongno-gu, Seoul, 03080, Republic of Korea, 82 2-740-8688; 2 Department of Medical Education and Humanities, College of Medicine, Yeungnam University, Daegu, Republic of Korea; 3 Department of Curriculum and Instruction, College of Education, University of Illinois, Urbana-Champaign, IL, United States; 4 Dental Research Institute, Seoul National University, Seoul, Republic of Korea; 5 Department of Family and Community Medicine, Macon and Joan Brock Virginia Health Sciences at Old Dominion University, Norfolk, VA, United States

**Keywords:** AI, artificial intelligence, competency-based education, curriculum, healthcare professionals, medical education, professional competence, qualitative research

## Abstract

**Background:**

As AI fundamentally transforms the healthcare landscape, medical education curricula have struggled to keep pace with these technological shifts. While current research has established a foundation for general AI literacy, less attention has been given to the role-specific competencies required for the diverse functions that healthcare professionals perform in an AI-integrated environment. Although existing tiered and domain-based competency models have clarified general AI competency requirements, they offer limited guidance on how such competencies should be differentiated according to the roles healthcare professionals perform in practice.

**Objective:**

This study aimed to explore how AI-driven changes in healthcare shape competency requirements across professional roles and to develop differentiated competency frameworks and curriculum implications for 3 distinct roles: users, developers, and leaders.

**Methods:**

Using a qualitative research design, we conducted in-depth interviews with 13 subject matter experts across academic medicine and dentistry, clinical practice, and the healthcare AI industry, who were recruited through 4 independent channels. Data were analyzed using reflexive thematic analysis with inductive coding organized around 3 research questions and published competency frameworks serving as sensitizing concepts. Trustworthiness was supported through investigator triangulation, member checking, an audit trail, and attention to researcher reflexivity and positionality.

**Results:**

We identified a role-differentiated competency framework with a layered structure as follows: (1) Users require machine learning and data literacy to understand where algorithms fail and scrutinize data origins and biases; the ability to select best-fit AI solutions and set appropriate thresholds for human-machine delegation; and a high degree of AI-related professionalism to work responsibly with AI by recognizing its limits, critically appraising outputs, maintaining clinical accountability, preserving patient-centered care, and updating knowledge continuously; (2) Developers add operational competencies, including validating systems across technical performance, clinical relevance, and regulatory compliance; bridging the language gap between clinical needs and technical refinements; and fostering a challenging, problem-solving mindset to address real-world clinical bottlenecks; and (3) Leaders focus on system-level competencies, including macro-level strategic planning, governing medical AI across its full lifecycle—selection, validation, deployment, and monitoring—through policy “roads” and governance frameworks, and optimizing systemic resource utilization while orchestrating trust-based collaboration that safeguards care quality. Rather than a strictly cumulative hierarchy, the roles represent analytic distinctions with shared foundational competencies. This framework translates these competencies into corresponding curriculum implications across the health professions education continuum.

**Conclusions:**

This study identifies role-differentiated AI competencies and proposes a corresponding curriculum scaffold for healthcare education. As a preliminary, hypothesis-generating starting point requiring multistakeholder validation, the proposed framework can help prepare the future healthcare workforce to utilize AI responsibly and to govern and lead the next generation of digital health innovation.

## Introduction

The rapid rise of AI is revolutionizing healthcare, fundamentally transforming traditional paradigms from automating routine administrative tasks to imaging-based diagnostic support, data-driven treatment personalization, and procedural assistance [1]. These advancements shift not only technological paradigms but also the fundamental roles and competencies expected of healthcare professionals. Medical professionals face the challenge of understanding AI and applying it responsibly in clinical contexts [2]. This creates an urgent need to restructure medical education to equip trainees with AI-related competencies.

This urgency contrasts with the slow pace of educational change [3,4]. Although AI is redefining medical competency requirements, many medical education institutions continue to adhere to traditional curricula. Several factors contribute to this lag. First, the systematic complexity and lengthy medical education cycles create a barrier for integrating new technology quickly. A second barrier is limited educator expertise in AI, which constrains the ability to translate it into teachable and assessable curricular content. As a result, medical education can trail healthcare innovation, limiting graduates’ readiness to evaluate and use AI responsibly.

To bridge this gap, clearly defining AI competencies provides shared, assessable outcomes that can guide curriculum design and scalable faculty development, enabling more systematic integration of AI beyond reliance on individual faculty expertise. Accordingly, recent studies have sought to define AI competencies in medical education. Garvey et al [5] and Lee et al [6] established core knowledge and ethical domains, while Moldt et al [7] and Russell et al [2] emphasized interdisciplinary assessment and continuous learning. Additionally, Schubert et al [8] proposed a tiered model ranging from basic to expert proficiency.

More recent efforts have broadened these discussions across general and health-specific contexts. The United Nations Educational, Scientific and Cultural Organization (UNESCO) developed separate AI competency frameworks for students and teachers, spanning domains such as human-centered values, ethics, AI foundations, applications, and pedagogy [9,10]. In medical education, the International Advisory Committee for Artificial Intelligence (IACAI) proposed recommendations for AI integration across individual, institutional, and system levels [11]. The Association of American Medical Colleges (AAMC) has articulated principles for responsible AI use [12] and is developing competencies across the continuum from undergraduate medical education (UME) through graduate medical education (GME) to continuing professional development (CPD) [13]. Building on these UNESCO and IACAI frameworks, Khamis et al [14] proposed a faculty development framework based on the Accreditation Council for Graduate Medical Education (ACGME) Milestones, with progression from basic AI literacy to leadership.

Collectively, these frameworks provide important foundations for AI competency development in health professions education. However, they generally organize competencies by educational stakeholder, level of expertise, training phase, or institutional implementation level, rather than by the functional responsibilities healthcare professionals assume in AI-enabled practice. Therefore, this study builds on these foundations by examining AI competencies across 3 functional roles—users, developers, and leaders—and translating them into curriculum implications across the health professions education continuum, thereby refining and extending existing tiered and domain-based models [8,15]. Although few graduates currently enter AI development or policy roles, demand for such professionals is likely to grow, necessitating a shift from generic literacy to role-aligned pathways.

This study aimed to explore the cascading impact of AI on healthcare and how these changes influence competency requirements across roles—from users to developers and leaders—ultimately informing the redesign of medical education curricula. The study is guided by three research questions: (1) How have AI advancements transformed healthcare practices? (2) What specific AI competencies are required for healthcare professionals across users, developers, and leaders? (3) What curriculum framework is needed to systematically cultivate these role-specific AI competencies?

## Methods

### Study Design

This study used a qualitative research methodology for an in-depth exploration of the complex perspectives of subject matter experts (SMEs) on AI-driven changes in healthcare and medical education, a rapidly evolving field where qualitative inquiry is essential to obtain contextually rich and at times competing expert views. The study is exploratory and hypothesis-generating in nature and is aimed at characterizing how experts conceptualize role-differentiated AI competencies in healthcare and medical education. We adopted reflexive thematic analysis as articulated by Braun and Clarke [16], a theoretically flexible interpretive approach in which themes are actively generated by the researcher through recursive engagement with the data. Reporting follows the Standards for Reporting Qualitative Research (SRQR) [17], and trustworthiness is addressed through the criteria proposed by Lincoln and Guba [18].

### Ethical Considerations

The study was approved by the Institutional Review Board of Seoul National University School of Dentistry (S-D20220028) and conducted in accordance with the Declaration of Helsinki. All participants provided written informed consent to participate in the study, including consent for audio recording and the use of anonymized excerpts in publications. Each participant received an honorarium of KRW 300,000 (approximately US $220) as compensation for their time and expertise.

### Participants

We operationally defined an SME as an individual meeting at least two of the following criteria: (1) a faculty position in a medical or dental school with documented research output on AI in healthcare or healthcare education; (2) active clinical practice integrating AI-based tools, evidenced by clinical leadership roles or published clinical work; (3) substantive operational involvement in an AI-related healthcare enterprise; and (4) recognized public visibility as a domain authority, evidenced by invited expert commentary in major national media.

Candidates were identified across the study period through four independent channels: (1) recommendations from 3 professional societies (the Korean Society of Artificial Intelligence in Medicine, the Korean Society of Medical Education, and the Korean Dental Education Association); (2) systematic screening of academic profiles at major medical and dental schools for AI-relevant research and curricular involvement; (3) identification through national daily news media of individuals featured as authoritative commentators on AI in healthcare; and (4) snowball recruitment, used principally to reach industry SMEs less visible through academic and media channels. We observed substantial convergence across these channels as society recommendations, academic searches, and media identifications repeatedly surfaced overlapping individuals, indicating recognized authority across independent professional and public spheres, which reduced the risk of bias from any single channel.

All approached experts agreed to participate, yielding 13 SMEs in total (Table 1). Several SMEs held dual or multiple roles (eg, academic faculty also engaged in clinical practice or in operating an AI-related healthcare enterprise), which we deliberately viewed as desirable during purposive selection because such individuals could provide broader perspectives to enrich the research questions.

**Table 1.** Professional backgrounds of SMEs^a^.

SME number	Professional role	Area of expertise	Degree	AI expertise				Interview phase^b^
				R^c^	C^d^	T^e^	D^f^	
1	Professor of medicine	Emergency medicine; AI clinical decision support	MD^g^, PhD^h^	✓	✓	✓	✓	Early^i^
2	Professor of medicine	Biomedical engineering; AI medical imaging	PhD	✓	—^j^	✓	✓	Early
3	Professor of medicine	Oncology/genomics; AI-based precision medicine	MD, PhD	✓	✓	✓	✓	Early
4	Professor of medicine	Medical AI; AI diagnostic imaging	PhD	✓	—	✓	✓	Early
5	Professor of medicine	Radiation oncology; AI clinical decision support	MD, PhD	✓	✓	✓	—	Mid^k^
6	Professor of dentistry	Medical informatics; medical ontology and machine learning	PhD	✓	—	✓	—	Late^l^
7	Professor of dentistry	Prosthodontics; AI-based dental CAD^m^ and workflow	DDS^n^, PhD	✓	✓	✓	✓	Early
8	Professor of dentistry	Prosthodontics; AI-assisted dental CAD and workflow	DDS, PhD	✓	✓	✓	✓	Mid
9	Dental practitioner	Prosthodontics/orthodontics; AI orthodontic diagnosis	DDS, PhD	✓	✓	—	✓	Late
10	Dental practitioner	Orthodontics; AI cephalometric analysis	DDS, PhD	✓	✓	—	✓	Mid
11	Healthcare AI company executive	Medical AI industry; AI-based cancer diagnostics	MD	✓	✓	—	✓	Late
12	Healthcare AI company executive	Dental AI industry; AI-based dental CAD	PhD	✓	—	—	✓	Mid
13	Healthcare AI company executive	Dental AI industry; AI medical/dental imaging	PhD	✓	—	—	✓	Early

a SME: subject matter expert.

b Interview phase reflects the initial interview.

c R: research indicates documented AI-related research output.

d C: clinical use indicates active clinical practice integrating AI-based tools.

e T: teaching indicates a faculty teaching role.

f D: development indicates substantive involvement in developing AI-based healthcare solutions or enterprises.

g MD: Doctor of Medicine.

h PhD: Doctor of Philosophy.

i Early: 2022‐2023.

j Not applicable.

k Mid: 2024.

l Late: 2025.

m CAD: computer-aided design.

n DDS: Doctor of Dental Surgery.

### Data Collection

A semistructured interview protocol was developed by 3 team members (YP, YH, and JL) and refined through written feedback from the remaining team members. The interview questions were organized around the 3 research questions (anticipated changes in healthcare, required AI-related competencies, and implications for future curricula) and deliberately framed broadly rather than anchored in any pre-existing competency taxonomy, allowing diverse expert perspectives to surface without being constrained by extant frameworks. To support the logical translation from competencies to curricular implications, participants were reminded of the competencies they had identified and asked to specify the curricular content needed to develop those competencies. No formal pilot was conducted; the first interview served as a calibration check and required no substantive revision to the protocol.

Of the 13 interviews conducted between October 2022 and October 2025, 12 were held in person and 1 was conducted via Zoom (Zoom Communications, Inc), with each interview lasting between 45 and 90 minutes. The interview phase in Table 1 reflects the initial interview. All interviews were audio-recorded with consent and transcribed verbatim.

Sample size was determined through iterative monitoring of analytic stabilization—sometimes termed data saturation, though we use this term cautiously given the constructivist orientation of reflexive thematic analysis [16]—rather than a prespecified target. By approximately the ninth interview, analytic discussions indicated that subsequent transcripts were generating few new codes and that emerging themes were being elaborated rather than expanded, suggesting that the dataset was approaching information sufficiency for the research questions. Four additional interviews were subsequently conducted to both verify this pattern and purposively probe emergent tensions that surfaced during data collection. These interviews yielded contextual elaborations rather than substantively new themes. By the 13th interview, the team judged the framework structure to have stabilized, with all 3 role categories sufficiently represented in the data. Sample adequacy was further evaluated against the information power framework [19], which judges qualitative sample sufficiency along 5 dimensions. Our study showed favorable indicators across four of these dimensions: (1) a focused research aim, (2) high participant specificity (each SME had expertise in a field directly relevant to the study), (3) substantive interview dialogue, and (4) a case-focused analytic strategy.

### Data Analysis

Interview data were analyzed using reflexive thematic analysis [16,20]. Rather than developing a deductive codebook prior to analysis, we organized inductive coding around the 3 research questions, with published competency frameworks identified during the literature review [2,6-8] serving as sensitizing concepts that provided theoretical guidance without imposing a predetermined analytic structure [21].

Data analysis began in October 2022 and proceeded iteratively alongside data collection. Following the 6 recursive phases of reflexive thematic analysis (data familiarization, generation of initial codes from the data, theme development, theme review, theme definition, and reporting) [20], 4 researchers (YP, YH, HK, and JL) independently coded the full dataset and convened iteratively to compare codes, discuss interpretive divergences, and refine candidate themes. During the theme review and definition phases, we specifically examined whether candidate themes reflected dentistry-specific examples or broader cross-specialty functions. Dentistry-specific examples, such as computer-aided design/computer-aided manufacturing (CAD/CAM)–supported treatment planning and procedure-specific digital design, were treated as illustrative rather than as sufficient grounds for defining general competencies. Themes were retained at the framework level when they reflected cross-cutting functions across dental, medical, engineering, and industry perspectives. Discrepancies that could not be resolved through team discussion were referred to 2 additional team members (SA and FWK) for adjudication. For cross-linguistic adjudication, JL translated relevant excerpts and contextual details into English. Quotations presented here were translated by JL and verified by FWK.

To ensure the accuracy of the interpretation, triangulations were carried out through member checking with all 13 SMEs in 2025. Each SME reviewed a summary of findings from their interview, either confirming that it reflected their views or providing updates or clarifications. Seven SMEs submitted detailed written feedback, which was incorporated into the analysis where relevant. Building on the competency themes refined through this process, we derived the curriculum implications by mapping participant-proposed educational content for each competency to the corresponding theme and, through iterative team discussion, organizing it into role-specific modules sequenced from foundational concepts to applied content.

The 6-member research team comprised researchers with multidisciplinary backgrounds spanning dental and medical education, educational technology, learning analytics, nursing practice in internal medicine settings, family medicine, and AI-enabled medical education technology. Four members based in Korean health professions education conducted coding (YP, YH, HK, and JL). Two members served as discrepancy resolvers (SA and FWK), with FWK additionally contributing a non-Korean clinical and technology-development perspective. We recognize that this composition concentrated our analytic gaze and that our shared identity as health professions education scholars predisposed us to read the data through a competency and curriculum lens. We treated these orientations as interpretive resources rather than biases to be eliminated [16], discussing them explicitly during analytic meetings to question candidate themes.

### Trustworthiness

Trustworthiness was addressed against the 4 criteria of Lincoln and Guba [18] as follows. *Credibility* was supported by prolonged engagement with the field across the data collection window, investigator triangulation through independent coding and discrepancy resolution, member checking with all 13 SMEs, and cross-linguistic adjudication of disputed interpretations. *Transferability* was supported through detailed description of recruitment context, participant characteristics, and analytic decisions; we explicitly note that the framework was developed within a Korean health professions education context with substantial dental representation, which may shape its applicability across healthcare systems, specialties, and regulatory environments. *Dependability* was supported by an audit trail comprising the interview protocol, transcripts, analytic memos, and codes developed iteratively during analysis. *Confirmability* was supported by the explicit positionality statement above, the use of verbatim participant quotations to ground interpretive claims, and the triangulation procedures noted above.

## Results

### Overview

We have presented the themes, subthemes, and competencies identified in this study. Moreover, we have provided selected quotes, and further quotes are available in Multimedia Appendix 1.

### Theme I: AI-Driven Healthcare Transformation

#### Overview

Thirteen SMEs described how AI integration is reshaping healthcare. Their perspectives anticipated four directions of change: (1) greater patient empowerment and more horizontal doctor-patient relationships; (2) redefinition and reconfiguration of medical expertise; (3) transformation and redesign of clinical workflows through human-machine collaboration; and (4) a possible shift toward more consistent “average” care quality through standardization and reduced operator variability, supporting routine clinical decisions and processes without displacing the judgment of skilled clinicians.

#### Subtheme I-1: Patient Empowerment and Horizontal Doctor-Patient Relationships

Experts suggested that patients will arrive with AI-generated interpretations and recommendations, reshaping the traditional information asymmetry. Clinicians may need to negotiate care decisions with patients who treat AI as an additional expert voice while critically assessing the clinical validity of AI outputs.

*Patients will become more empowered… “The AI says something’s off, so why do you think it’s fine?”… patients now have knowledge that makes them almost equal to clinicians.*

[SME #1]

*Once patients view AI as an expert service, it’s like bringing another doctor along… “This is a second opinion from a well-known AI.”*

[SME #3]

#### Subtheme I-2: Redefinition and Reconfiguration of Medical Expertise

SMEs expected AI to blur and reorganize professional boundaries by shifting tasks across specialties and to AI systems (SMEs 1, 3, 5, and 8). Some also anticipated a deeper reconfiguration of expertise, shifting from fixed territories to data-driven, high-level integration (SMEs 3 and 5).

*I think the boundaries of healthcare professionals’ duties will need to be redefined—literally redefined. The tasks that used to be considered their exclusive responsibilities may have to be redefined in this new context.*

[SME #8]

*With AI, the boundaries… start to blur. Fields that used to fight over territory will naturally merge or shift as data and algorithms provide objective evidence… AI will accelerate this shift, lowering the walls between specialties and redefining what expertise means.*

[SME #5]

*I think physicians’ roles will actually become more advanced. It’s like when you no longer have to focus on keeping your lane on the highway—you can … focus on other, more important things. Similarly, doctors will rely on AI to handle the detailed tasks and instead concentrate on broader, higher-level judgments.*

[SME #1]

#### Subtheme I-3: Transformation of Clinical Workflows

Experts expected that AI would fundamentally reshape medical workflows by reallocating tasks between humans and machines. They emphasized that this transformation would depend on the effective integration of human-machine collaboration models.

*If we think of all the tasks… as 100%, about 80% could eventually be handled by AI… Humans should then focus on the remaining 20%… Ultimately, how well we build the human-machine collaboration model will shape everything… AI will fundamentally transform medical workflows.*

[SME #3]

Another expert illustrated this potential operational efficiency, noting that AI could streamline the logistics of consultations and referrals. By reducing unnecessary steps in the referral process, AI might allow workflows to proceed more quickly, potentially enabling clinicians in some settings to allocate more time to direct patient interaction.

*With AI, instead of asking five people, you might only need to ask two or three. … You can quickly figure out who to refer to, so the whole referral process becomes smoother. What used to take a full round of visits or a week to follow up now happens much faster—the workflow just flows better.*

[SME #5]

#### Subtheme I-4: Improvement in Average Healthcare Quality

Finally, experts expected that AI could improve the consistency of routine care by reducing operator variability, even if it does not replace the craftsmanship of exceptional clinicians.

*AI can’t match the craftsmanship of what we create by hand—one stitch at a time. It will never reach the high end.*

[SME #9]

*It’s not that the maximum level of care will go up, but the average will… local clinics… could reach the average quality level of university hospitals.*

[SME #8]

*Before AI, the quality used to vary a lot… But with AI, those operator differences have gotten much smaller.*

[SME #5]

Taken together, the findings of research question 1 indicate that AI-driven change operates across relational, professional, workflow, and care quality dimensions of healthcare. These dimensions provide the contextual basis for the role-differentiated competencies described below.

### Theme II: Role-Differentiated AI Competencies and Curriculum Design

#### Overview

Experts agreed that the competencies and educational needs of medical students should differ depending on their future roles: those who will primarily use AI in clinical practice, those who will participate in AI development, and the small group of future leaders who will drive innovation and governance in the field of medical AI.

*Out of ninety students, maybe two or three will be truly inspired by an AI course and become the ones who lead this country—and even compete globally—in medical AI. For the majority, though, the goal should be to understand how to position themselves within the AI context and use it effectively in practice. Such a dual structure is essential, especially in a cutting-edge field like this.*

[SME #2]

To clarify the boundaries among the competencies, the framework differentiates roles according to their primary function and level of responsibility in AI-enabled healthcare. We have defined each role operationally. AI users apply AI tools in routine clinical practice—interpreting AI-generated outputs, integrating them with clinical judgment, and remaining professionally accountable for patient care. AI developers design, evaluate, or contribute to building AI solutions—translating clinical needs into technical specifications or working within clinical-AI development teams. AI leaders govern, oversee, or strategically direct AI integration at organizational or systemic levels, for example, department heads, chief medical informatics officers, and healthcare administrators overseeing institutional AI implementation.

These roles describe positions in current or future clinical practice, and the curriculum continuum (UME, GME, and CPD) is designed to prepare learners for them. They are analytic distinctions rather than mutually exclusive paths. Many SMEs held dual roles, and the framework is best understood as layered: each role carries a distinct emphasis, while foundational competencies (eg, critical evaluation of AI outputs and accountability for clinical judgment) are shared across roles. The distribution across roles is also asymmetric: most healthcare professionals will serve as users, with smaller numbers entering developer or leader roles.

#### Subtheme II-1: AI User Competencies and Curriculum Design

##### Competency U-1: AI/Machine Learning Literacy

Most SMEs endorsed AI and machine learning (ML) literacy as a baseline competency for future users—a practical understanding of learning-based AI mechanisms sufficient to understand what AI does, where it can fail, and why errors occur.

*Algorithms are like engines—you don’t need to develop them, but you should know enough to understand when they might fail. With a car, I can feel it—it shakes or slows down—but AI doesn’t feel like that. You just input software and get results. That’s why we need more education on the underlying mechanisms.*

[SME #1]

However, some argued that detailed ML instruction is not essential for all students and should mainly serve to inspire potential future developers.

*Doctors don’t need to learn machine learning itself… if a few get inspired… that’s already a success.*

[SME #3]

##### Competency U-2: Data Literacy

Data literacy was repeatedly framed as indispensable, as AI outputs reflect underlying data biases. SMEs emphasized the need to scrutinize data origins, labeling processes, and potential institutional biases.

*Data literacy is essential… datasets vary widely… results can differ because of how data are collected and interpreted.*

[SME #1]

*In one large chest X-ray dataset, only about a thousand of a hundred thousand images had actually been reviewed by physicians, and even those were flawed. It shows how important it is to verify and understand what kind of data are being used.*

[SME #2]

Across both AI/ML literacy and data literacy, SMEs stressed that users must be able to interpret and explain outputs meaningfully to patients, rather than merely operate tools, underscoring this as a critical mandate for medical education (SMEs 2, 3, and 11).

*Using an AI solution isn’t difficult—it’s as easy as using Microsoft Word. The real challenge is understanding what the output implies and how to apply it meaningfully in practice. That’s what education should address.*

[SME #3]

##### Competency U-3: AI Solution Selection and Utilization

Building on foundational AI/ML and data literacy, SMEs emphasized the ability to select and manage AI tool use in practice: understanding AI solutions, comparing options, anticipating bias, and setting thresholds for when to rely on AI versus human expertise.

*AI solutions will continue to diversify, and each one will have its own type of data bias. Physicians will face situations where they must decide which solution best fits a given patient.*

[SME #1]

Consequently, experts urged that education must teach how to set these critical thresholds—determining precisely when to rely on automation versus human expertise (SMEs 3 and 4).

*In radiology, for instance, general practitioners can now interpret many imaging results directly using AI software, referring only ambiguous or difficult cases to radiologists. This means that doctors can internalize some of the radiologist’s expertise while using AI for efficiency. Education should teach how to set such thresholds—when to rely on AI and when to rely on human expertise.*

[SME #3]

Some experts questioned the necessity of explicit AI training, arguing that solid domain knowledge is the true prerequisite for validating AI outputs.

*In prosthodontics… if those basics are neglected, they won’t be able to tell whether an AI-generated design aligns with fundamental dental principles.*

[SME #7]

However, others countered this by stating that while foundational mastery is vital, its very definition evolves.

*After giving a lecture on digital technology, students often ask, “Why are we still using wax and torches? Shouldn’t we be doing digital practice?” I tell them, you must understand the fundamentals first—but I also think the fundamentals themselves are changing… Education needs to reflect those updated fundamentals.*

[SME #8]

##### Competency U-4: AI Professionalism in Healthcare

SMEs identified AI professionalism as a core competency, which can be organized into 4 aspects.

First, experts noted that a foundational element of AI professionalism is an awareness of AI’s inherent limitations, that is, its outputs are bounded by prior data and cannot adapt on their own to changing clinical conditions, and that education must address this explicitly.

*AI is just a tool—and that’s something every doctor needs to remember… When medical guidelines or systems change, it might start doing things that make no sense… it will always be limited by what it has learned from past data. Students must be clearly taught about these limits.*

[SME #4]

*When AI makes an unexpected decision, it’s not really an error—it’s just doing what it was taught. Understanding that helps physicians communicate better, make fewer mistakes, and keep patients safer.*

[SME #4]

Second, experts emphasized that physicians must uphold ethical accountability and remain the final arbiters of clinical judgment, and education should treat this as a top priority.

*There are already cases of AI misuse—overreliance, really… Education must repeatedly stress that, in the end, any mistake or harm remains the physician’s responsibility. That’s what it means to hold a medical license.*

[SME #4]

*AI systems can assist in diagnosis, but they don’t make the diagnosis… If we delegate that responsibility to AI, we risk giving away our very professionalism.*

[SME #10]

*It’s about helping students understand the gravity of working with AI… They need to know where their responsibility begins and ends… in practice.*

[SME #1]

Third, experts highlighted the importance of preserving the human dimensions of care—understanding the patient’s context and showing empathy—as distinctly physician responsibilities.

*Seeing the patient, understanding their context, and showing empathy—those are things only a physician can do. That’s what needs to be taught.*

[SME #11]

Fourth, SMEs further argued that this professionalism must be sustained through lifelong learning. They noted that because AI tools, evidence, and regulations evolve quickly, maintaining up-to-date understanding—and developing the ability to critically interpret new research—should be treated as part of professional integrity.

*Being good at using tools right away doesn’t make you a good doctor. What really matters is continuous learning—it’s a core part of a physician’s role and responsibility.*

[SME #1]

Synthesizing these expert perspectives, AI professionalism may be defined as the physician’s ability to work ethically and responsibly with AI systems by recognizing AI’s inherent limitations, critically evaluating AI outputs against the clinical context while accepting accountability as the final arbiter of clinical decisions, integrating AI use with empathic patient-centered care as a distinctly human responsibility, and continually updating knowledge amid the rapid evolution of AI evidence and regulations.

Table 2 outlines the curriculum implications aligned with AI user competencies, aligning competencies (U-1 to U-4) with educational modules. The progression moves from foundational literacy to applied selection and professionalism, guiding learners from basic understanding to responsible use.

**Table 2.** Curriculum implications aligned with AI user competencies.

Required competencies	Educational content
U-1. AI/ML^a^ literacy: Understanding AI mechanisms to identify where algorithms fail and why errors occur	Module 1. Introduction History of AI Changes in healthcare driven by AI adoption AI ecosystem: social and infrastructural perspectives
U-2. Data literacy: Scrutinizing data origins and biases to explain AI outputs meaningfully to patients	Module 2. AI/ML and data literacy AI/ML fundamentals Data literacy
U-3. AI solution selection and utilization: Selecting best-fit AI tools and setting thresholds for human-machine delegation	Module 3. AI solution understanding, selection, and utilization Understanding and selection of AI solutions Application of AI in healthcare Human-AI collaboration
U-4. AI professionalism in healthcare: Maintaining accountability as final arbiters of clinical judgment through lifelong learning	Module 4. AI-related professionalism Limits of AI Roles, accountability, and ethics of healthcare professionals Lifelong learning

a ML: machine learning.

### Subtheme II-2: AI Developer Competencies and Curriculum Design

#### Competency D-1: Advanced User Foundations

SMEs described developer competency as building on user competencies with advanced ML literacy and professional awareness. Developers must understand how data quality; rigorous annotation, including expert-defined labels and richer clinical metadata, clinically defined reference standards such as lesions or diagnoses, and decision thresholds for clinical risk; and interpretability underpin trustworthy medical AI (SME 13). This includes understanding how model outputs can be interpreted in relation to the training data, how different data features shape model judgments, and what additional clinical data may be needed to improve performance. Developers must also recognize that their technical decisions directly impact clinical trust, patient safety, and social accountability (SMEs 1 and 11).

*In the end, developing an algorithm is really all about the data… the annotation process—how you label and define the correct answers—needs to be done well… With explainable AI (XAI), you can look at the output and think, “Okay, this model made this judgment based on that data—so maybe if we include more of this kind of data, the performance will improve.” That kind of understanding is really valuable.*

[SME #13]

#### Competency D-2: Effectiveness Evaluation

Experts emphasized that developers must evaluate AI systems across 3 critical dimensions: technical performance, clinical relevance, and regulatory compliance. What distinguishes AI evaluation in healthcare from evaluation in other engineering domains is that each dimension carries healthcare-specific implications. Regarding technical performance, experts noted that developers should understand how clinical trial results form the “credible evidence that can later be used for reimbursement and policy decisions” (SME 11), and education must go beyond accuracy metrics to cover validation processes. Crucially, developers must interpret performance thresholds in clinical contexts, adjusting sensitivity-specificity tradeoffs according to screening, diagnostic, or workflow priorities.

*Developers shouldn’t just read the model’s performance table; they must be able to judge its clinical value in different medical settings. For instance, in breast cancer screening, sensitivity matters most—even at the cost of more false positives—while in chest X-rays, that trade-off could disrupt the workflow. Developer training should cover how to interpret such contextual differences and adjust sensitivity–specificity targeting.*

[SME #11]

For clinical relevance, developers must learn to weigh medical necessity against technical feasibility and judge whether model performance translates into meaningful clinical value in specific medical settings.

*Engineering often starts from… “Can we make it?” But medicine must also ask, “Should we use it?” ... Developers must learn to weigh clinical benefit against technical possibility.*

[SME #1]

For regulatory compliance, the curriculum must bridge the gap between research models and commercial products.

*Regulatory expectations are extremely high… There’s a huge gap between research-grade models and commercially approved ones… Developer education should… train students to understand international regulatory systems.*

[SME #11]

This requires developers to assess compliance within constantly evolving legal and institutional frameworks (SME 10).

*Because these legal and institutional contexts evolve, developers must go beyond technical design and learn to assess whether their systems remain compliant within the current regulatory framework.*

[SME #10]

#### Competency D-3: Field Demand Communication

Experts stressed that effective clinical-technical communication between clinicians and developers is essential for ensuring AI addresses genuine clinical needs. Developers must learn to understand contextual differences across institutions and translate clinical language into technical refinements, engaging in continuous dialogue throughout the development process. This communication challenge is particularly complex in healthcare because, even within the same institution, different specialties may define the same clinical condition differently.

*Developers shouldn’t look at hospital data only in statistical terms—they need to understand the real clinical problems behind it. Even within one hospital, pulmonologists and thoracic surgeons may define the same condition differently. Learning to communicate and reconcile these differences is key.*

[SME #1]

*Clinicians and developers speak completely different languages… Clinicians talk about clinical meaning, while developers talk about accuracy scores… education that bridges that gap…*

[SME #10]

*Over time, clinicians get a sense for when the AI will fail or succeed, and the product improves through that feedback… ongoing dialogue…*

[SME #4]

#### Competency D-4: Challenging Mindset and Problem-Solving

Experts emphasized that developers must cultivate curiosity and perseverance to tackle real clinical problems through experimentation, reflection, and iterative improvement. Education should facilitate the resolution of real-world clinical bottlenecks.

*When radiologists said, “We’ve been trying to solve this problem for years…” I took on the challenge… within a month, the performance improved dramatically. That kind of problem-solving experience… really motivates both clinicians and developers.*

[SME #4]

*A spirit of challenge is essential… It’s about curiosity… The mindset that says, “What if we make that happen?”—that’s what education should nurture.*

[SME #9]

Table 3 outlines the curriculum implications aligned with AI developer competencies, aligning competencies (D-1 to D-4) with educational modules. The progression moves from advanced technical mastery to evaluation, communication, and innovation, fostering both engineering precision and clinical adaptability.

**Table 3.** Curriculum implications aligned with AI developer competencies.

Required competencies	Educational content
D-1. Advanced user foundations: Advanced foundational skills—ML^a^/data literacy, selection, and professionalism—within the developer role, with attention to clinically grounded data and model interpretation	Module 1. Advanced user competency Advanced understanding of ML/data literacy and AI solution use Expanded perspectives on AI-related developer professionalism Clinical data quality, expert annotation, clinical reference standards, and model-output interpretation
D-2. Effectiveness evaluation: Validating systems across technical performance, clinical relevance, and regulatory compliance in healthcare settings	Module 2. Evaluation of AI in healthcare Technical validation and performance evaluation of AI systems Clinical effectiveness assessment Policy and regulatory compliance review Clinical trial evidence, safety demonstration, and reimbursement/policy relevance
D-3. Field demand communication: Bridging the “language gap” to translate clinical needs into technical refinements, including specialty-specific clinical definitions	Module 3. Field communication and feedback Identifying clinical needs and collecting feedback for AI solutions Enhancing collaboration through shared language and understanding Reconciling specialty-specific clinical definitions and workflow priorities
D-4. Challenging mindset and problem-solving: Applying curiosity and perseverance to solve real-world clinical bottlenecks	Module 4. Innovation Developing a proactive and innovative mindset Problem-solving in complex and dynamic healthcare contexts

a ML: machine learning.

### Subtheme II-3: AI Leader Competencies and Curriculum Design

#### Competency L-1: Systemic Insight and Strategic Planning

Experts emphasized that AI leadership starts with macro-level sensemaking: leaders must anticipate how AI reshapes workflows and infrastructures beyond mere automation.

*An AI leader isn’t just someone who holds a position—it’s someone with the vision… to set the direction… [and] draw the big picture…*

[SME #5]

*I saw the massive wave of industrial transformation approaching, … The moment plaster models became digital scans, the entire pipeline—treatment, manufacturing, and business—was revolutionized… Students today must be educated to recognize and anticipate such paradigm shifts in the industry.*

[SME #9]

Crucially, this vision must align with social readiness rather than just technical feasibility.

*The key question isn’t whether a technology can be realized, but whether society is ready… AI leadership education must address… social acceptance, ethical judgment, and systemic policy awareness.*

[SME #8]

*AI leadership requires the capacity to plan beyond technology—to design interconnected infrastructures, workforce systems, and policies that support these changes. Education for AI leaders must therefore cultivate systemic thinking, policy implementation skills, and integrated management of resources and human capital.*

[SME #3]

Finally, experts stressed collaborative execution and operational efficiency as essential qualities for AI leaders. The ability to coordinate across clinical, technical, and administrative teams determines the success of integration in practice (SME 9).

#### Competency L-2: AI Governance and Policy Design

Unlike developers who focus on evaluating the performance of individual AI systems, leaders are expected to evaluate AI at the governance and policy level, integrating regulations, infrastructure, and accountability frameworks.

*Drugs, for instance, go through Phases 1 to 4, but applying the same staged framework to AI doesn’t really fit. We need a more suitable regulatory model for AI applications in medicine.*

[SME #1]

Experts highlighted that leadership competency involves planning AI policy and infrastructure roadmaps that enable coordinated ecosystem growth. While developers optimize algorithms, leaders must design the “roads” that sustain them, integrating data systems, institutions, and national strategies.

*It’s like we keep trying to make better cars, but no one’s building the roads…*

[SME #6]

Experts further emphasized that governance must operate across the entire AI lifecycle, beginning with tool selection and procurement. Leaders must evaluate clinical feasibility, usability, and workflow fit rather than technical capability alone, and—where regulatory agencies cannot adjudicate—judge an algorithm’s soundness at the institutional level.

*With 30 clinical departments each saying “ours matters,” the hospital itself has to judge whether an algorithm is sound—much as the regulatory agency judges a device.*

[SME #1]

They noted that technical capability alone does not guarantee real-world adoption and that procurement decisions must therefore weigh whether a system will actually be used in practice.

Watson started from engineering… but ended up not being used at all. You can build it, but whether it gets used is another matter.

[SME #1]

Beyond adoption, experts stressed that leaders must institutionalize internal validation, recognizing that vendor evidence often reflects controlled conditions rather than real clinical performance.

*When you give it simulation cases, it looks helpful… but that’s in vitro. It hasn' t yet been witnessed in vivo, in actual clinical application.*

[SME #1]

At the deployment stage, they framed implementation not as installation but as an integration effort requiring coordination across clinical and infrastructure teams.

*When applying AI in clinical environments, customizing the setup to each hospital’s context is crucial… involving IT [information technology] teams, EMR [electronic medical record] administrators, and support departments… and designing multidisciplinary governance structures.*

[SME #9]

Finally, experts underscored that governance extends into postdeployment monitoring, since AI is trained on past data and degrades as clinical guidelines and environments shift, requiring continuous oversight of bias, off-label use, and overreliance.

*Each solution carries bias in different ways, and the clinician has to choose among them for each patient.*

[SME #1]

*The more important part is that it has to be continuously updated.*

[SME #4]

These risks ultimately return to accountability: when a system drifts, errs, or is overrelied upon, governance must already have defined who bears responsibility—ethical, legal, and operational—across developers, planners, and users.

*In medical systems, we need to clearly define who holds responsibility, risk, and transparency. What are the roles of developers, planners, and users? How should accountability be shared among them? These questions must guide national-level AI governance frameworks and should be explicitly addressed in AI leadership education.*

[SME #6]

#### Competency L-3: Resource Management and Leadership

Experts emphasized that AI leaders must manage healthcare resources at a systems level rather than compromising patient-level care quality (SME 1), institutionalize organization-wide task allocation rules based on contextual complexity to build trust-based human-machine collaboration (SME 3), and support implementation through cross-role communication and workflow-adjustment mechanisms linking clinical teams with AI developers and maintainers (SME 8).

*The goal is… reducing systemic waste… AI can function as a tool to optimize resource utilization… Future healthcare leaders should be trained… from this systems-oriented perspective.*

[SME #1]

*Building systems where AI supports, but doesn’t replace, human expertise… humans manage the complex… machines take over structured, predictable tasks… That’s how we build trust.*

[SME #3]

*Communication is the hardest part… it won’t work if the users can’t communicate effectively with those who build or maintain it… when schedules or workflows need to be adjusted… human dialogue and coordination remain irreplaceable.*

[SME #8]

Table 4 outlines the curriculum implications aligned with AI leader competencies (L-1 to L-3), mapping each competency to educational modules. The progression expands from macro-level strategic planning to governance design and resource management, fostering capabilities for system-wide transformation and collaborative execution.

**Table 4.** Curriculum implications aligned with AI leader competencies.

Required competencies	Educational content
L-1. Systemic insight and strategic planning: Leading macro-level sensemaking and vision-setting for AI-driven workflow and infrastructure shifts in healthcare	Module 1. Systems thinking and strategic planning Reading macro trends and planning system-level changes Recognizing social context and applying systems approaches Enhancing collaboration and operational efficiency in hospital AI implementation
L-2. AI governance and policy design: Evaluating medical AI at the governance and policy level, designing AI policy “roads,” and governing the full AI lifecycle	Module 2. AI governance and policy design Regulation and evaluation of AI applications in medicine Policy and infrastructure roadmap planning Designing lifecycle governance (tool selection/procurement → validation → deployment → continuous monitoring/accountability)
L-3. Resource management and leadership: Optimizing systemic resource utilization and orchestrating trust-based collaboration while safeguarding care quality	Module 3. Resource management and leadership Resource optimization and healthcare supply-chain management aligned with care quality and workflow coordination Human–machine collaboration and trust-based leadership Team communication and collaborative leadership

**Figure 1.**
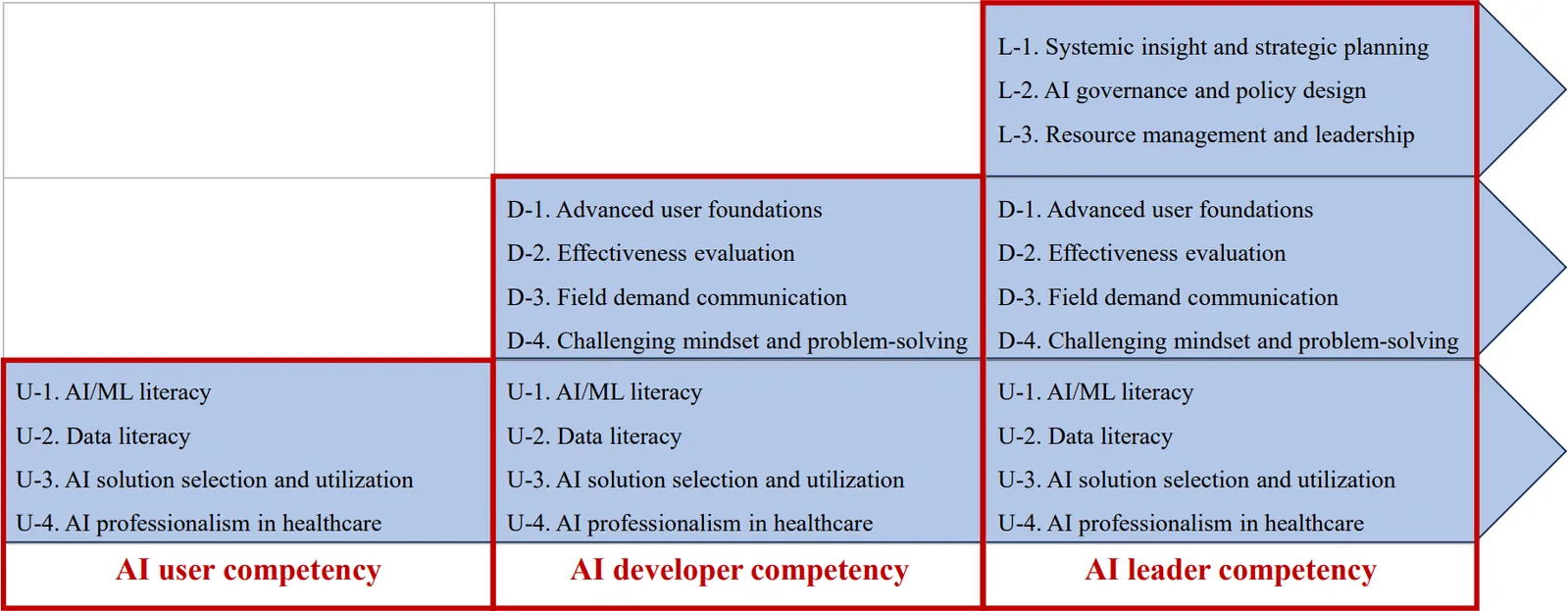
Role-differentiated AI competency framework across user, developer, and leader roles.

Figure 1 illustrates the role-differentiated AI competency framework across AI users, developers, and leaders. It visualizes the layered structure in which each role carries a distinct emphasis—foundational AI literacy and clinical judgment for users, operational and technical translation for developers, and system-level governance for leaders—while shared foundational competencies provide common ground across roles.

## Discussion

### Principal Findings

**Figure 2.**
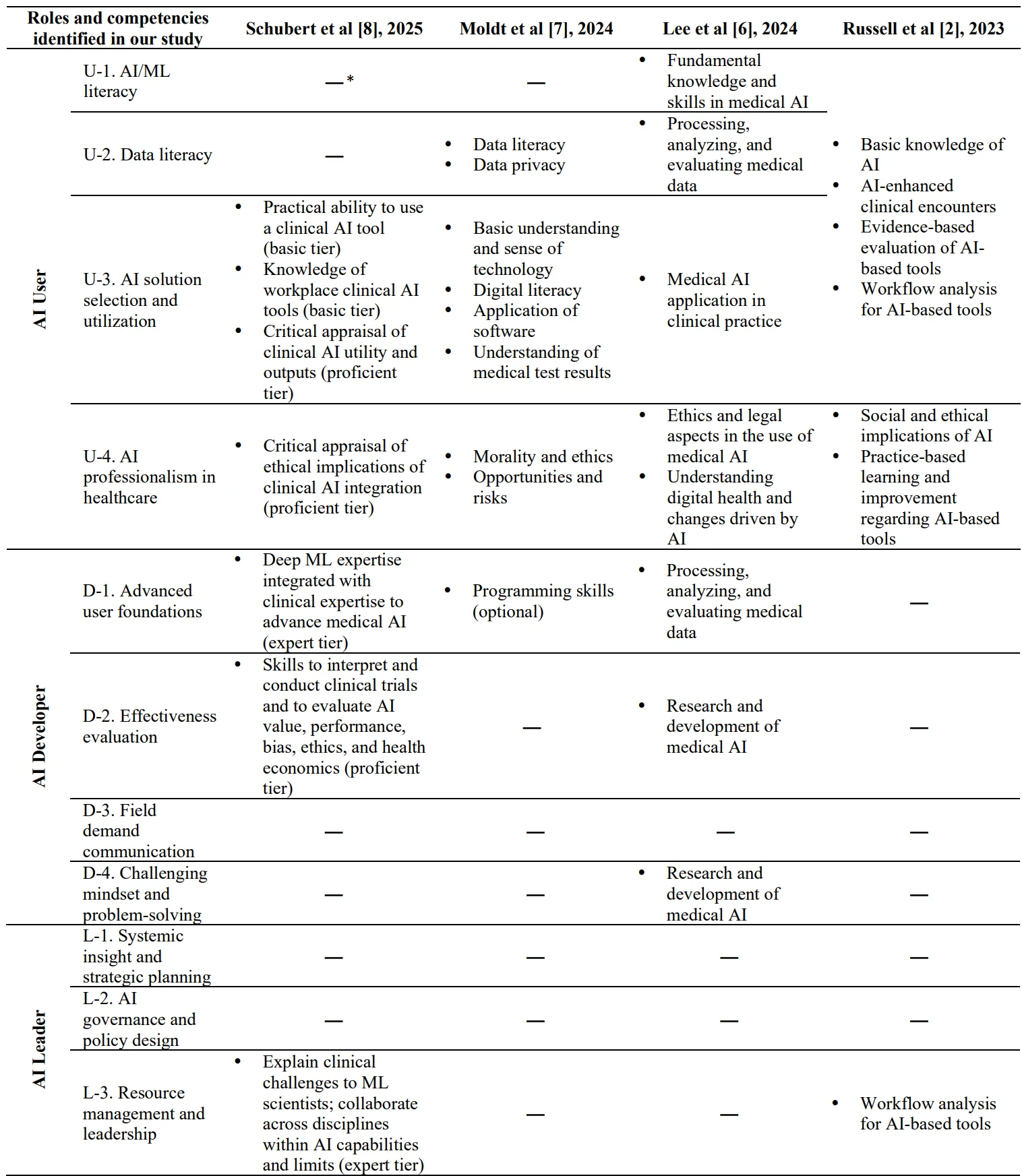
Comparison of competencies identified in this study and the recent literature [2,6-8]. ML: machine learning. *Not reported.

This study explored AI-driven changes in healthcare, identified role-differentiated AI competencies for healthcare professionals (AI users, developers, and leaders), and mapped these competencies to corresponding educational modules as curriculum implications. The framework is best understood as a layered structure with role-specific emphases and shared foundational competencies. To further contextualize our findings in relation to existing AI competency frameworks, Figure 2 presents an interpretive comparison rather than a formal validation or analytic procedure. This comparison clarifies how the proposed role-differentiated framework may extend and reorganize existing competency domains through a functional role lens.

Regarding AI-driven changes in healthcare, experts anticipated four interrelated directions: (1) greater patient empowerment and more horizontal doctor-patient relationships, (2) redefinition and reconfiguration of medical expertise, (3) transformation of clinical workflows through human-machine collaboration, and (4) improvement in average healthcare quality through reduced operator variability. These projections, however, should be understood less as settled futures than as conditional and contested propositions.

Patient empowerment may enhance participation in care, yet patient-facing AI can also generate fluent but inaccurate interpretations that patients may be poorly positioned to evaluate. A recent case study in clinical neuropsychology showed that patient-led use of a large language model misrepresented clinical data, caused distress, and increased clinical burden by requiring direct correction of AI-generated misinformation [22]. Similarly, physicians report both perceived benefits and concerns regarding AI in medical decision-making and patient-physician communication, underscoring that AI may be supportive but may also strain clinical relationships when trust, accountability, and AI literacy are insufficient [23]. The shift toward more consistent average care is likewise double-edged: while AI may reduce unwarranted variation and raise the floor of care, it may also anchor clinical practice to a merely acceptable average, reinforce automation bias, and contribute to the erosion of independent expertise [24-26]. This risk is supported by a multicenter observational study showing that endoscopists’ adenoma detection rate during non–AI-assisted colonoscopy declined after routine exposure to AI-assisted colonoscopy, providing real-world evidence of AI-associated deskilling in relation to a clinically meaningful outcome [25]. The redistribution of expertise and redesign of workflows likewise remain open questions rather than inherently beneficial developments: AI may augment clinical decision-making and support more patient-centered workflows [27], but it may also fragment professional identity [28], diffuse accountability [29], and shift tasks according to technical feasibility rather than clinical value unless implementation is guided by human-centered design, governance, and clearly defined clinical oversight [30].

Figure 2 shows that recent literature has largely prioritized user-level competencies for medical students and practicing health professionals [2,6-8]. Across these studies, the most consistent emphasis is on competencies U-3 and U-4, reflecting a broad consensus that clinicians must interpret and apply AI outputs responsibly, communicate their implications, and maintain professional and ethical accountability for clinical decision-making. Existing frameworks offer valuable multilevel and system-based guidance but are less often organized around healthcare professionals’ functional responsibilities in AI-enabled practice [2,6-8,11,13]. From this role-based perspective, sustainable AI integration requires user competencies alongside specialized developer competencies (eg, validation design, translation of clinical needs, and safety and bias checks) and leader competencies (eg, governance design, accountability structures, resource allocation, and strategic decision-making).

The present framework does not replace existing efforts but complements them by adding a functional-role perspective. Rather than organizing competencies by educational stakeholder type, expertise level, training phase, or implementation level, it organizes them around what healthcare professionals do with AI—using it in clinical care, contributing to its development, or governing it at organizational and system levels. The user-developer-leader structure is therefore a pragmatic, simplified, role-focused analytic framework for curriculum design, not a linear hierarchy or a set of mutually exclusive career pathways. This simplification reflects the distributed, context-dependent nature of AI-enabled healthcare, in which responsibilities overlap across individual, team, institutional, and policy levels. For example, educators may function as users when applying AI in teaching and assessment, as developers when co-designing educational AI systems, or as leaders when shaping institutional policies. Thus, the framework extends existing multilevel models while recognizing that individuals may move across or combine roles depending on context.

This study revealed an unresolved disagreement about the appropriate scope, depth, and educational priority of AI-related competencies across the health professions education continuum. Notably, experts’ perspectives appeared to vary according to their professional backgrounds and practical experiences with AI. Experts from academia with AI research involvement more often discussed AI as a transformative force reshaping healthcare workflow, professional boundaries, and the distribution of expertise (SMEs 1, 2, 6, and 8), whereas experts in procedurally intensive fields emphasized foundational hands-on expertise and the preservation of human clinical judgment (SMEs 7, 10, and 12). At the same time, an expert familiar with digital transformation suggested that the very notion of what is considered foundational or fundamental is itself evolving in the AI era (SME 8). Meanwhile, experts with industry and development experience frequently highlighted implementation, communication, and governance issues across clinical and technical domains (SMEs 4, 11, and 13). Thus, the controversy was not simply whether specific content should be included, but how to balance technological preparedness with foundational professional mastery. Nevertheless, we draw a tentative conclusion that a minimal level of ML and data literacy should be included, consistent with the predominant expert view and patient-safety imperatives [31-34]. While rapid AI advances and shifting clinical workflows may reshape what constitutes necessary content, curricular debates should remain anchored in enduring professional functions [2,5,6].

**Figure 3.**
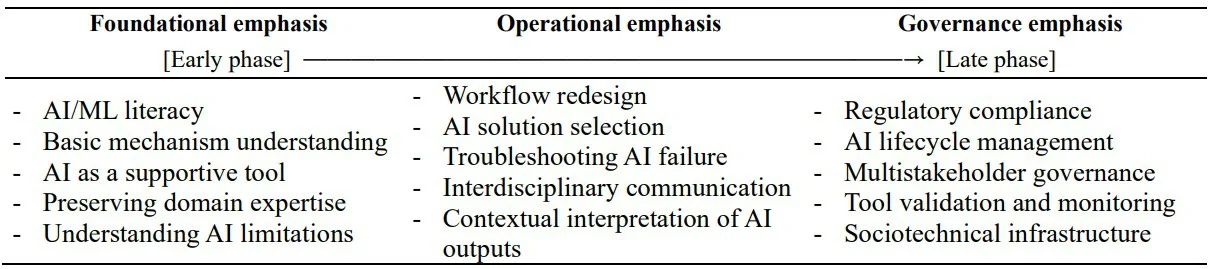
Temporal distribution of expert perspectives (early vs late period, 2022-2025). ML: machine learning.

The interview window extended over 3 years (October 2022 to October 2025), spanning the public emergence of large language models in clinical workflows. Comparing early- and late-period codes showed that the emphasis of expert concerns shifted—foundational issues dominated earlier interviews, while operational and system-level considerations were elaborated in greater specificity later (Figure 3; verbatim quotations by phase are provided in Multimedia Appendix 2). Later framings did not replace earlier ones: foundational concerns about the limits of AI and clinical judgment anchored expert reasoning throughout, and later interviews introduced no new role categories. This temporal consistency, despite a rapid change in the field, supports the durability of the role-differentiated framework, with content within each role, particularly at the system level, continuing to develop.

Operationalizing a role-based framework raises questions about what to teach and where—across UME, GME, and CPD—to cultivate these competencies. Our findings revealed expert disagreement over whether detailed AI/ML instruction is necessary for all learners, suggesting that curricular implementation should remain context-sensitive rather than uniform across institutions. Institutions may embed user competencies as required UME content while offering developer and leader competencies through elective or advanced pathways, including physician-scientist pathways such as MD-PhD programs [35-37]. Conversely, emphasizing GME/CPD may mitigate UME crowding and leverage clinical experience [38,39], while hybrid sequencing can maintain continuity across stages [40,41]. The balance between core and elective content across UME, GME, and CPD will depend on institutional mission, learner trajectories, and faculty expertise.

To make the curriculum implications more actionable, assessment should also be aligned with the role-differentiated and layered structure of the framework. Because several competencies identified in this study are dispositional, practice-based, or systems-oriented rather than purely knowledge-based, they are unlikely to be adequately assessed through written examinations alone. Instead, they may be better evaluated through programmatic assessment that draws on multiple data points and complementary methods [42,43]. For user competencies, AI professionalism could be assessed through objective structured clinical examination–style stations or reflective documentation in which learners interpret an AI-generated recommendation, identify potential limitations or biases, communicate uncertainty to a patient, and document their final clinical decision and accountability rationale. For developer competencies, a challenging mindset and problem-solving could be assessed through simulation-based failure-mode tasks in which learners analyze why an AI tool performs poorly in a given clinical workflow, propose iterative refinements, and justify safety and bias-mitigation checks. For leader competencies, systemic insight could be assessed through case-based governance simulations or policy briefs that require learners to map stakeholders, anticipate implications for workflow and accountability, and design monitoring and escalation procedures. These assessment examples should be regarded as illustrative strategies rather than validated instruments; future work should develop rubrics and collect validity evidence across UME, GME, and CPD contexts.

Faculty readiness, raised by several experts, represents a further barrier to implementation [41,44]. Most current health professions educators were trained before AI became central to clinical practice, and few have direct experience integrating AI into teaching. Studies suggest that institutions must pursue short-term interinstitutional collaborations, including jointly developed faculty training programs, shared AI curriculum modules, and AI educator fellowships, to rapidly build a critical mass of qualified teaching faculty across institutions. A recent study has demonstrated the feasibility of structured, theory-informed faculty development workshops on integrating generative AI in medical education [45]. In parallel, long-term organizational readiness and systematic faculty development, supported by hybrid teaching models that pair clinical educators with AI specialists, have been proposed as a means to build sustainable internal capacity [40,46,47].

Beyond faculty readiness, implementation requires flexible adaptation of the framework across specialty contexts. Dentistry served as an information-rich case because AI applications are increasingly visible across dental clinical practice, including image interpretation, treatment planning, digital design, CAD/CAM-supported workflows, and procedural decision support [48-50]. These applications illustrate how AI can reshape clinical tasks, human-machine delegation, and professional responsibility. By contrast, in less procedure-oriented or less image-intensive specialties and care contexts, such as psychiatry, internal medicine, primary care, or longitudinal chronic care, the same role functions may be expressed through different AI applications, including risk prediction, documentation, triage, longitudinal decision support, patient communication, or population management. Thus, the framework should be understood as a set of transferable role functions that require specialty-specific operationalization, rather than as a model specific to dentistry.

### Limitations and Future Research Recommendations

The user-developer-leader framework proposed in this study was derived from a limited number of expert interviews situated largely within a single national context in East Asia. Accordingly, the findings should be interpreted with attention to the healthcare delivery system, reimbursement structure, and national regulatory and AI policy landscape (including AI governance and data-privacy requirements). Because these requirements differ across jurisdictions—for example, between this context and frameworks, such as the Health Insurance Portability and Accountability Act (HIPAA), General Data Protection Regulation (GDPR), and European Union (EU) AI Act—the proposed model is best understood as an adaptable template rather than a one-size-fits-all approach.

In addition, because several dental experts were included in the sample, the findings may reflect AI use cases that are more visible in imaging-intensive, CAD/CAM-supported, and procedure-oriented workflows. Their transferability may therefore vary in specialties or care contexts where AI is used more for longitudinal reasoning, narrative documentation, relational care, risk prediction, or population-level management. To address these boundaries, we applied explicit SME selection criteria and incorporated nondental clinical, medical education, engineering-related, and industry perspectives; nevertheless, transferability across other contexts and specialties remains to be empirically established.

Lastly, interrater reliability was not formally assessed, limiting quantitative evidence of coding consistency. Although this study, as a qualitative interpretive inquiry, aims to achieve transferability—supported by multiple trustworthiness procedures—rather than statistical generalizability or consensus-based validation, additional work is needed to strengthen the empirical grounding and translational utility of the proposed framework. We therefore envision several subsequent validation phases: (1) a modified Delphi process—consistent with structured, multiphase approaches to AI competency development, such as the AAMC’s ongoing initiative to define competencies across the medical education continuum [13]—involving broader multistakeholder participation, including clinicians, learners, patients, educators, regulators, policymakers, and institutional leaders across diverse healthcare systems and specialties; (2) pilot curriculum implementation studies to evaluate feasibility, acceptability, and educational outcomes; and (3) iterative refinement informed by multi-institutional feedback and longitudinal evaluation. Future research should also translate these role-differentiated AI competencies into measurable learning outcomes and develop assessment approaches appropriate to users, developers, and leaders. Such work may help determine how AI competencies can be evaluated longitudinally across health professions curricula, rather than relying solely on a single knowledge-based assessment.

### Conclusions

Despite the growing integration of AI into clinical practice, concrete guidance remains limited regarding which competencies physicians using AI should develop, what levels of proficiency are required, and which educational approaches are most appropriate [51]. In this context, our role-differentiated framework—and its translation into curriculum implications across the medical education continuum—offers a preliminary, hypothesis-generating starting point for institutions to make these decisions more explicit, to begin aligning training with real-world responsibilities, and to address the risk of diffused accountability in AI-enabled clinical care. In this way, adopting a role-based orientation in medicine may support physicians in remaining the “human-in-the-loop,” serving as the moral and intellectual center of patient care.

## Supplementary material

10.2196/97608 Multimedia Appendix 1 Additional illustrative quotes from subject matter experts mapped to themes and role-differentiated competency domains (users, developers, and leaders).

10.2196/97608 Multimedia Appendix 2 Verbatim quotations supporting Figure 3, tagged by interview phase.

## Abbreviations

AAMC: Association of American Medical Colleges
ACGME: Accreditation Council for Graduate Medical Education
CAD: computer-aided design
CAM: computer-aided manufacturing
CPD: continuing professional development
GDPR: General Data Protection Regulation
GME: graduate medical education
HIPAA: Health Insurance Portability and Accountability Act
IACAI: International Advisory Committee for Artificial Intelligence
ML: machine learning
SME: subject matter expert
SRQR: Standards for Reporting Qualitative Research
UME: undergraduate medical education
UNESCO: United Nations Educational, Scientific and Cultural Organization
