# Supplementary material for: Role-Differentiated AI Competencies and Curriculum Implications for Health Professions Education: Qualitative Study
Source: JMIR Med Educ. 2026 Sep 8;12:e97608. doi: 10.2196/97608 (PMC13552830; doi:10.2196/97608)
Supplement: Supplementary file 1 [file mededu-v12-e97608-s001.docx]

| **Multimedia Appendix 1.** Additional illustrative quotes from subject matter experts mapped to themes and role-differentiated competency domains (users, developers, and leaders). | | |  |
| --- | --- | --- | --- |
| **Theme / Competency** | **Illustrative Quotes from SMEs** | |  |
| **I. AI-driven Healthcare Transformation** | | **Analytic Linkage** | **Interview**  **phase** |
| **I-1. Patient Empowerment and Horizontal Doctor–Patient Relationships** | Patients will become more empowered. They’ll say, ‘According to this advanced AI, nothing’s wrong—why are you worried?’ or the opposite— ‘The AI says something’s off, so why do you think it’s fine?’ In that sense, patients now have knowledge that makes them almost equal to clinicians. (SME 1) | Potentially greater patient agency and reduced information asymmetry | Early |
|  | Once patients view AI as an expert service, it’s like bringing another doctor along— ‘This is a second opinion from a well-known AI. Could you explain why it differs from yours?’ The doctor–patient relationship has traditionally been vertical, but while patients once came armed with research papers, coming in with up-to-date AI-generated opinions will be another story. (SME 3) | Possible positioning of AI as an additional expert voice | Early |
| **I-2. Redefinition and Reconfiguration of Medical Expertise** | I think the boundaries of healthcare professionals’ duties will need to be *redefined* —literally redefined. The tasks that used to be considered their exclusive responsibilities may have to be redefined in this new context. (SME 8) | Potential redefinition of professional roles and task boundaries | Mid |
|  | Surgery is done by surgeons, and chemotherapy by oncologists. But Watson for Oncology was the one telling doctors which chemotherapy would work best. The moment a surgeon looks at the AI’s recommendation and says, ‘Okay, that makes sense—let’s go with that,’ the oncologist’s expertise starts to get taken over. That’s how this kind of *redistribution* of professional roles will happen. (SME 3) | Possible redistribution of specialty expertise and redefinition of professional role boundaries | Early |
|  | I think physicians’ roles will actually become *more advanced*. It’s like when you no longer have to focus on keeping your lane on the highway—you can look around and focus on other, more important things. Similarly, doctors will rely on AI to handle the detailed tasks and instead concentrate on broader, higher-level judgments. (SME 1) | Potential shift toward higher-level clinical judgment | Early |
|  | With AI, the boundaries of expertise will start to blur. Fields that used to fight over territory—like psychiatry and neurology with dementia—will naturally merge or shift as data and algorithms provide objective evidence. In the end, power will come from who can build stronger evidence: designing large studies, collecting data, and producing results. AI will accelerate this shift, lowering the walls between specialties and redefining what expertise means. (SME 5) | Potential blurring and reconfiguration of specialty boundaries | Mid |
| **I-3. Transformation of Clinical Workflows** | If we think of all the tasks in a given medical field as 100%, about 80% could eventually be handled by AI with nearly 99.9% accuracy. Humans should then focus on the remaining 20%—the parts that truly require human attention and judgment. As this cycle continues, more tasks will gradually be delegated to AI, and workflows will keep evolving toward greater efficiency and specialization. Ultimately, how well we build the human–machine collaboration model will shape everything—from hospital systems to physicians’ practice. AI will fundamentally transform medical workflows. (SME 3) | Potential human–AI task redistribution | Early |
|  | You know how we usually ask around—‘maybe that person knows?’ or ‘should I ask someone else?’ With AI, instead of asking five people, you might only need to ask two or three. It makes consultations way more efficient. You can quickly figure out who to refer to, so the whole referral process becomes smoother. What used to take a full round of visits or a week to follow up now happens much faster—the workflow just flows better. (SME 5) | Potential streamlining of consultation and referral workflows | Mid |
| **I-4. Improvement in Average Healthcare Quality** | It’s not that the maximum level of care will go up, but the average will. You can’t replace the truly exceptional doctors, but those in the middle tier—like local clinics—could reach the average quality level of university hospitals. (SME 8) | Potentially more consistent routine care quality | Mid |
|  | Before AI, the quality used to vary a lot depending on the resident’s skill or condition that day. But with AI, those operator differences have gotten much smaller, and the results stay more consistent. (SME 5) | Potential reduction in operator-dependent variability | Mid |
|  | AI can’t match the craftsmanship of what we create by hand—one stitch at a time. It will never reach the high end. (SME 9) | Potential limits of AI in replacing expert craftsmanship | Late |
|  | What really matters is that a medical license alone doesn’t guarantee ongoing expertise. That’s why continuing education is important—but AI is even more reliable. It can help maintain the quality of care by drawing on the input of countless experts and supporting professional competence. (SME 3) | Possible support for maintaining care quality and professional competence | Early |
| **II-1. AI User Competencies and Curriculum Design** | | **Analytic Linkage** | **Interview**  **phase** |
| **U-1. AI/ML Literacy** | Understanding basic ML concepts is becoming part of AI literacy. As AI becomes common in daily life—especially in medicine—students should at least know what it is. (SME 4) | Potential baseline AI/ML literacy | Early |
|  | We’re like professional drivers, but we don’t build cars ourselves. Algorithms are like engines—you don’t need to develop them, but you should know enough to understand when they might fail. With a car, I can feel it—it shakes or slows down—but AI doesn’t feel like that. You just input software and get results. That’s why we need more education on the underlying mechanisms. (SME 1) | Potential understanding of AI mechanisms and failure points | Early |
|  | Doctors don’t need to learn machine learning itself. For most, understanding data is enough. Still, if a few get inspired and move into AI development, that’s already a success. (SME 3) | Potentially appropriate depth of AI/ML literacy for general users | Early |
|  | When I was in medical school, I took a course on machine learning, and it really inspired me. Even though I didn’t plan to pursue it then, it kept influencing my interests later on.” (SME 10) | Possible early exposure inspiring future AI-related interests | Mid |
| **U-2. Data Literacy** | Data literacy is essential for anyone using AI. AI is built on data, but datasets vary widely across institutions and populations. Even with the same definitions, the results can differ because of how data are collected and interpreted. (SME 1) | Potential attention to data provenance, variability, and interpretation | Early |
|  | There’s a lot of bias in how we collect data. We don’t ask what we’re not curious about, and those gaps affect how AI learns and then feeds back into practice. Students need to understand this clearly when they use AI. (SME 1) | Potential data collection bias and downstream effects | Early |
|  | In one large chest X-ray dataset, only about a thousand of a hundred thousand images had actually been reviewed by physicians, and even those were flawed. It shows how important it is to verify and understand what kind of data are being used. (SME 2) | Potential need for data quality and labeling scrutiny | Early |
|  | When an algorithm identifies a target or lesion, future physicians don’t need to know every detail of how it was trained. However, they must understand what the results represent, what those findings mean, and be able to explain them meaningfully to patients. (SME 11) | Potential clinical interpretation of AI outputs | Late |
|  | Using an AI solution isn’t difficult—it’s as easy as using Microsoft Word. The real challenge is understanding what the output implies and how to apply it meaningfully in practice. That’s what education should address. (SME 3) | Potential emphasis on output interpretation and meaningful clinical application | Early |
| **U-3. AI Solution Selection and Utilization** | AI is basically an IT solution. They need to distinguish between the AI platform itself and the software solutions developed on that platform. For example, Watson is IBM’s AI platform, and Watson for Oncology is a solution built on it. AI solutions isn’t much different from Microsoft Word or PowerPoint, except that it provides a certain degree of medical expertise—partly, not fully. That’s the right way to see it. (SME 3) | Possible distinction between general platforms and clinical AI solutions | Early |
|  | AI solutions will continue to diversify, and each one will have its own type of data bias. Physicians will face situations where they must decide which solution best fits a given patient. (SME 1) | Potential selection of patient-specific AI solutions | Early |
|  | To diagnose and treat patients properly, you ultimately need to understand which tool is the best one. (SME 4) | Potentially appropriate tool selection | Early |
|  | We must know what kinds of expertise can be enhanced through AI and what areas remain uniquely human. To stay competitive, physicians should strengthen the expertise that AI cannot easily replace, while also leveraging AI to expand their competence. (SME 3) | Potential identification of AI-complemented expertise and uniquely human expertise | Early |
|  | In radiology, for instance, general practitioners can now interpret many imaging results directly using AI software, referring only ambiguous or difficult cases to radiologists. This means that doctors can internalize some of the radiologist’s expertise while using AI for efficiency. Education should teach how to set such thresholds—when to rely on AI and when to rely on human expertise. (SME 3) | Potential human–AI delegation thresholds | Early |
| **Controversies** | Not everyone needs to learn machine learning or data literacy. While understanding these concepts can certainly help individuals excel in their field, they are not mandatory skills for most professionals. Just like we use smartphones or computers without knowing how they work internally, AI will be the same. What really matters is having strong domain knowledge to judge whether AI’s results make sense. Doctors don’t need to build AI—they need to interpret and evaluate its output using their expertise. Of course, advanced users or future developers might need that deeper technical knowledge, but for the majority, it’s not essential. (SME 5) | Potential role of domain knowledge in judging AI outputs | Mid |
|  | I’m not sure training on how to use AI is even necessary. Do we need special training to ride in a self-driving car? (SME 1) | Possible questioning of the need for dedicated operational training | Early |
|  | I don’t think any special skill is required to use AI. It’s as easy as using Word or PowerPoint. What matters is how you use it. (SME 3) | Potential emphasis on meaningful use over technical operation | Early |
|  | In prosthodontics, students often skip manual wax-ups and focus only on CAD design. But hands-on practice is essential. If those basics are neglected, they won’t be able to tell whether an AI-generated design aligns with fundamental dental principles. Comparing AI outputs with one’s own foundational knowledge is crucial. (SME 7) | Potential evaluation against clinical principles | Early |
|  | We need to educate physicians on what to do when AI fails. In digital dentistry, for instance, if a problem can’t be solved with advanced technology, you must know how to revert to earlier methods—even manual ones. Understanding that flow helps you judge whether a limitation comes from lack of knowledge or from the tool itself. (SME 12) | Potential need for fallback strategies when AI tools fail | Mid |
|  | After giving a lecture on digital technology, students often ask, ‘Why are we still using wax and torches? Shouldn’t we be doing digital practice?’ I tell them, you must understand the fundamentals first—but I also think the fundamentals themselves are changing. What was fundamental before may no longer be the same today. Education needs to reflect those updated fundamentals. (SME 8) | Potential updating of clinical fundamentals in AI use | Mid |
| **U-4. AI Professionalism in Healthcare**  **U-4. AI Professionalism in Healthcare** *(Con’t)* | AI is just a tool—and that’s something every doctor needs to remember. AI is always based on the past. When medical guidelines or systems change, it might start doing things that make no sense. Unless AI can autonomously sense changes, update itself, and collect new data—which it can’t—it will always be limited by what it has learned from past data. Students must be clearly taught about these limits. (SME 4) | Potential awareness of AI limitations | Early |
|  | When AI makes an unexpected decision, it’s not really an error—it’s just doing what it was taught. Understanding that helps physicians communicate better, make fewer mistakes, and keep patients safer. AI isn’t technically perfect, and education needs to make that clear. (SME 4) | Potential understanding of data-bound AI behavior and safety | Early |
|  | AI can tell you that a lesion exists, but it can’t judge how clinically serious it is. That’s where the doctor’s role comes in—integrating what AI shows with the patient’s symptoms, lab results, history, and overall condition. You can’t just believe AI’s output as it is. Seeing the patient, understanding their context, and showing empathy—those are things only a physician can do. That’s what needs to be taught. (SME 11) | Potential integration of AI outputs with patient context | Late |
|  | There are already cases of AI misuse—overreliance, really. The FDA recently warned against using AI not designed for triage as a triage tool. Some doctors skip reviewing ‘normal’ cases because the AI said they were fine, or just sign off automatically. That’s ethically problematic. Education must repeatedly stress that, in the end, any mistake or harm remains the physician’s responsibility. That’s what it means to hold a medical license. (SME 4) | Potential accountability and overreliance prevention | Early |
|  | AI systems can assist in diagnosis, but they don’t make the diagnosis. They simply indicate measurement points or suggest patterns. The dentist must still confirm the findings and establish the treatment plan. If we delegate that responsibility to AI, we risk giving away our very professionalism. This principle should be strongly emphasized in the curriculum. (SME 10) | Potential preservation of final clinical judgment | Mid |
|  | It’s about helping students understand the gravity of working with AI—how it actually operates, when it might go wrong, and when they, as future physicians, need to step in and make critical decisions. They need to know where their responsibility begins and ends, and how to carry that weight in practice. (SME 1) | Potential clarification of professional responsibility boundaries | Early |
|  | You don’t need to understand AI at an engineer’s level. What matters is knowing how it works, how it’s regulated, and what its limits are—and then keeping that knowledge up to date. The real problem is that many universities teach the same course, the same materials, year after year. In AI, that means what you learned last year could already be obsolete. (SME 4) | Potential ongoing awareness of AI limits and regulation | Early |
|  | The goal isn’t just to train people who can quickly use the latest tools. Forgetting a phone number doesn’t mean you’re bad at relationships—it’s the same idea. Being good at using tools right away doesn’t make you a good doctor. What really matters is continuous learning—it’s a core part of a physician’s role and responsibility. (SME 1) | Potential framing of lifelong learning as professional responsibility | Early |
|  | In an AI course, I think we should keep things fairly light. We can’t really dig too deep in a dental and medical school context. It would be enough to cover some basic, liberal-arts-level content—like the history of AI, how it’s been developed. (SME 9) | Potential foundational awareness for responsible AI use | Late |
|  | It would be better to cover conceptual aspects—like the basic idea of AI, case studies showing how AI is being applied in medical practices, and discussions about where and how it might be used in the future. Personally, I find even the conceptual background, such as the history and evolution of AI, really interesting on its own. (SME 10) | Potential conceptual understanding for responsible AI use | Mid |
|  | There’s no need to go too deep into the subject because the field itself changes so quickly. […] About 130 AI tools have been approved so far, and most medical schools already have introductory courses that help students become aware of what’s available. Knowing what’s out there helps them identify which tools they might want to try using. (SME 4) | Potential ongoing awareness of evolving AI tools | Early |
| **II-2. AI Developer Competencies and Curriculum Design** | | **Analytic Linkage** | **Interview**  **phase** |
| **D-1. Advanced User Foundations** | In the end, developing an algorithm is really all about the data. When you build it on reliable data, the algorithm’s performance naturally improves. So, first, the raw data itself has to be good, and second, the annotation process—how you label and define the correct answers—needs to be done well. Both are equally important parts of the process. These days, we talk about explainable AI, or XAI^[1]^. It’s basically a way for the system to show the reasoning behind its decisions. If you understand how that works, you can look at the output and think, ‘Okay, this model made this judgment based on that data—so maybe if we include more of this kind of data, the performance will improve.’ That kind of understanding is really valuable. (SME 13) | Potential emphasis on data quality, annotation, and model interpretation | Early |
| **D-2. Effectiveness Evaluation** | In practice, I often take part in clinical trial design, reporting, and result interpretation. These results ultimately form the credit evidence that can later be used for reimbursement and policy decisions. Developers should understand this whole process. (SME 11) | Potential evidence generation for reimbursement and policy decisions | Late |
|  | Engineering often starts from the question, ‘Can we make it?’ But medicine must also ask, ‘Should we use it?’ An AI solution could be technologically impressive but clinically irrelevant. Developers must learn to weigh clinical benefit against technical possibility. (SME 1) | Potential clinical value beyond technical feasibility | Early |
|  | Developers shouldn’t just read the model’s performance table; they must be able to judge its clinical value in different medical settings. For instance, in breast cancer screening, sensitivity matters most—even at the cost of more false positives—while in chest X-rays, that trade-off could disrupt the workflow. Developer training should cover to interpret such contextual differences and adjust sensitivity–specificity targeting. (SME 11) | Potential context-specific clinical value and trade-off judgments | Late |
|  | Regulatory expectations are extremely high. Many AI products introduced on the market still fail to meet these thresholds. There’s a huge gap between research-grade models and commercially approved ones. To be implemented in hospitals, performance and safety must be demonstrated conservatively, and the requirements vary by country. Developer education should address this reality and train students to understand international regulatory systems. (SME 11) | Potential regulatory and safety requirements | Late |
|  | Because these legal and institutional contexts evolve, developers must go beyond technical design and learn to assess whether their systems remain compliant within the current regulatory framework. (SME 10) | Potential changes in compliance requirements | Mid |
| **D-3. Field Demand Communication** | When we create AI products, we constantly exchange feedback with clinicians—especially in large hospitals. …Over time, clinicians get a sense for when the AI will fail or succeed, and the product improves through that feedback. Learning how to engage in that kind of ongoing dialogue should be part of developer education. (SME 4) | Potential continuous clinical feedback | Early |
|  | Developers shouldn’t look at hospital data only in statistical terms—they need to understand the real clinical problems behind it. Even within one hospital, pulmonologists and thoracic surgeons may define the same condition differently. Learning to communicate and reconcile these differences is key. (SME 1) | Potential translation of clinical problems into technical refinements | Early |
|  | In our collaborations with companies, one of the biggest challenges is that clinicians and developers speak completely different languages. Clinicians talk about clinical meaning, while developers talk about accuracy scores. We need education that bridges that gap and teaches both sides how to communicate effectively. (SME 10) | Potential bridging of clinical and technical language | Mid |
| **D-4. Challenging Mindset and Problem-Solving** | When radiologists said, ‘We’ve been trying to solve this problem for years with no success,’ I took on the challenge. I got the data through the proper process, and within a month, the performance improved dramatically. That kind of problem-solving experience—of identifying a bottleneck and fixing it—really motivates both clinicians and developers. (SME 4) | Potential problem-solving around clinical bottlenecks | Early |
|  | If you understand when AI fails to operate properly, you can anticipate and prevent errors. For example, AI can’t perform well in dark environments, just like a camera can’t recognize faces in low light. So education should teach troubleshooting when and why AI may malfunction, rather than just how it works internally. (SME 6) | Potential troubleshooting of AI malfunctions | Late |
|  | A spirit of challenge is essential for AI developers. It’s not just about coding—it’s about curiosity. The best developers are the ones who see a new technology and wonder, ‘Could this be possible?’ Remember Minority Report, when Tom Cruise manipulated floating screens? That was science fiction once, but now it’s real. The mindset that says, ‘What if we make that happen?’—that’s what education should nurture. (SME 9) | Potential curiosity and innovation mindset | Late |
| **II-3. AI Leader Competencies and Curriculum Design** | | **Analytic Linkage** | **Interview**  **phase** |
| **L-1. Systematic Insight and Strategic Planning**  **L-1. Systematic Insight and Strategic Planning** *(Con’t)* | An AI leader isn’t just someone who holds a position — it’s someone with the vision and philosophy to set the direction. The details can be filled in by the team, but the leader needs to draw the big picture and guide others with a clear, thoughtful roadmap. (SME 5) | Potential vision-setting and strategic direction | Mid |
|  | One of the most essential capabilities for an AI leader is the ability to view technology not merely as an automation tool but as a force that reshapes the entire industrial workflow. (SME 3) | Anticipated system-level workflow change | Early |
|  | I used to be a cautious, stability-oriented dentist who quietly focused on patient care. But when I saw the massive wave of industrial transformation approaching, I couldn’t stay still. I realized that relying solely on clinical practice had limits, and that we needed to translate our clinical expertise into digital products. The moment plaster models became digital scans, the entire pipeline—treatment, manufacturing, and business—was revolutionized. […] Students today must be educated to recognize and anticipate such paradigm shifts in the industry. (SME 9) | Potential transformation of workflow, infrastructure, and industry structures | Late |
|  | The key question isn’t whether a technology can be realized, but whether society is ready to accept it. For example, telemedicine isn’t limited by technology but by real-world readiness in some countries. This example shows that AI leadership education must address not only technical excellence but also social acceptance, ethical judgment, and systemic policy awareness.” (SME 8) | Potential social readiness and policy judgment | Mid |
|  | For instance, in logistics automation, it’s not just about vehicles driving themselves—it’s about redesigning the division of labor between humans and systems. […] In this context, AI leadership requires the capacity to plan beyond technology—to design interconnected infrastructures, workforce systems, and policies that support these changes. Education for AI leaders must therefore cultivate systemic thinking, policy implementation skills, and integrated management of resources and human capital.” (SME 3) | Potential planning of infrastructure, workforce, and policy | Early |
| **L-2. AI Governance and Policy Design** | When it comes to AI solutions, we don’t yet have a clear regulatory framework. Drugs, for instance, go through Phases 1 to 4, but applying the same staged framework to AI doesn’t really fit. We need a more suitable regulatory model for AI applications in medicine. (SME 1) | Potential need for AI-specific regulatory and governance models | Early |
|  | When applying AI systems in clinical environments, customizing their setup to fit each hospital’s context is crucial. It’s not only a physician’s concern but also a collaborative process involving IT [information technology] teams, EMR [electronic medical record] administrators, and support departments. The success of AI integration depends on how effectively these stakeholders coordinate as a system. Thus, the ability to design and manage multidisciplinary governance structures should be a key component of AI leadership education. (SME 9) | Potential context-sensitive implementation and stakeholder coordination | Late |
|  | With 30 clinical departments each saying 'ours matters,' the hospital itself has to judge whether an algorithm is sound—much as the regulatory agency judges a device. (SME 1) | Potential institutional evaluation of AI soundness | Early |
|  | Watson started from engineering… but ended up not being used at all. You can build it, but whether it gets used is another matter. (SME 1) | Potential real-world adoption judgment | Early |
|  | When you give it simulation cases, it looks helpful… but that's in vitro. It hasn't yet been witnessed in vivo, in actual clinical application. (SME 1) | Potential real-world clinical validation | Early |
|  | The more important part is that it has to be continuously updated. (SME 4) | Potential continuous updating | Early |
|  | Each solution carries bias in different ways, and the clinician has to choose among them for each patient. (SME 1) | Potential bias-aware AI selection | Early |
|  | Even as time passes, there still aren’t enough experts to develop a true AI roadmap. The problem is that everyone focuses on machine learning itself—AI has become one-legged, so to speak. It’s like we keep trying to make better cars, but no one’s building the roads. A great engine alone doesn’t make a car run; the ecosystem needs coordination between infrastructure, hardware, and design. (SME 6) | Potential AI policy roadmap and ecosystem coordination | Late |
|  | In medical systems, we need to clearly define who holds responsibility, risk, and transparency. What are the roles of developers, planners, and users? How should accountability be shared among them? These questions must guide national-level AI governance frameworks and should be explicitly addressed in AI leadership education. (SME 6) | Potential accountability, risk, transparency, and role allocation | Late |
| **L-3. Resource Management and Leadership** | Resource and supply chain management is a critical issue in hospitals. Human resources, equipment, and beds are all directly linked to costs, and optimizing their use is essential. There are two levels to consider: first, reducing costs at the patient level, since patients make the initial decision to use medical services and generally wish to minimize hospital visits; and second, reducing costs at the system level. Because profit margins in healthcare are limited, the goal is not simply cost-cutting for individual patients—which could harm care quality—but reducing systemic waste. Ultimately, AI can function as a tool to optimize resource utilization across hospitals and healthcare systems, rather than focusing solely on the individual patient level. Future healthcare leaders should be trained to understand resource management from this systems-oriented perspective. (SME 1) | Potential system-level resource optimization while maintaining care quality | Early |
|  | As with autonomous vehicles, the challenge isn’t always technical capability but human trust. In cities, people still hesitate to accept driverless cars, even though they may be statistically safer. But on highways, the trust dynamic changes. I often describe this as a human–machine collaboration model: humans manage the complex, context-sensitive urban environments, while machines take over structured, predictable tasks such as highway driving. This is the same mindset we need in healthcare—building systems where AI supports, but doesn’t replace, human expertise. That’s how we build trust. (SME 3) | Potential trust-based task allocation | Early |
|  | In engineering, some people know nothing about medicine, while others have collaborated enough to understand clinical principles and terminology. Likewise, some clinicians understand the logic of engineering. The key is continued interaction between these two groups. People who share both languages—medical and technical—are the ones who can really make things happen. Education should therefore create opportunities for engineers and clinicians to meet, discuss, and experiment together, translating ideas into tangible outcomes. (SME 7) | Potential clinical–technical interaction and shared understanding | Early |
|  | Honestly, communication is the hardest part. No matter how fancy the software is, it won’t work if the users can’t communicate effectively with those who build or maintain it. For example, when schedules or workflows need to be adjusted, algorithms might automatically allocate resources—but clinicians often want flexibility that the system doesn’t yet support. That’s where frustration arises. Future dentists need to learn how to communicate effectively across roles—technicians, hygienists, engineers—because when problems occur, human dialogue and coordination remain irreplaceable. (SME 8) | Potential cross-role communication for workflow coordination | Mid |

1. Explainable Artificial Intelligence (XAI): a diverse set of methods aimed at rendering the decisions and predictions of AI models understandable to human users (Ciardiello et al., 2026).

   Ciardiello A, D'Angelo A, De Angelis L, Giagu S, Sala E, Gigante G. Beyond the black box: lessons in explainability from AI in mammography. Artificial Intelligence Review. 2026;59(5):130. doi:10.1007/s10462-026-11518-5.
