# Supplementary material for: Role-Differentiated AI Competencies and Curriculum Implications for Health Professions Education: Qualitative Study
Source: JMIR Med Educ. 2026 Sep 8;12:e97608. doi: 10.2196/97608 (PMC13552830; doi:10.2196/97608)
Supplement: Supplementary file 2 [file mededu-v12-e97608-s002.docx]

**Multimedia Appendix 2.** Verbatim quotations supporting Table 6, tagged by interview phase

Of the 61 analytic linkages related to role-based AI competencies and curriculum design presented in Multimedia Appendix 1, 38 (62.3%) were identified in the early-phase interviews, 11 (18.0%) in the mid-phase interviews, and 12 (19.7%) in the late-phase interviews. User-level linkages were concentrated primarily in the early phase, whereas 9 of the 12 linkages identified in the late phase (75.0%) pertained to the Developer or Leader levels. This pattern suggests that operational and system-level considerations were articulated with greater specificity over time. The quotations below, tagged by interview phase (Early = October 2022–2023, Mid = 2024, Late = 2025; Table 1), illustrate that all three emphases in Table 6 were voiced across the interview window.

| **Foundational emphasis** | **Operational emphasis** | **Governance emphasis** |
| --- | --- | --- |
| [Early phase] ―――――――――――――――――――――――→ [Later phase] | | |
| - AI/ML literacy  - Basic mechanism understanding  - AI as a supportive tool  - Preserving domain expertise  - Understanding AI limitations | - Workflow redesign  - AI solution selection  - Troubleshooting AI failure  - Interdisciplinary communication  - Contextual interpretation of AI outputs | - Regulatory compliance  - AI lifecycle management  - Multi-stakeholder governance  - Tool validation & monitoring  - Sociotechnical infrastructure |
| SME **1**, **2**, **3**, **4**, 5, **7**, **13** | SME 1, 3, 4, **5**, **8**, **10**, **12** | SME 1, 3, 5, **6**, 8, **9**, **11** |
| “Algorithms are like engines—you don’t need to develop them, but you should know enough to understand when they might fail. With a car, I can feel it—it shakes or slows down—but AI doesn’t feel like that. You just input software and get results. That’s why we need more education on the underlying mechanisms.” (SME 1, Early)  “In one large chest X-ray dataset, only about a thousand of a hundred thousand images had actually been reviewed by physicians, and even those were flawed. It shows how important it is to verify and understand what kind of data are being used.” (SME 2, Early)  “We must know what kinds of expertise can be enhanced through AI and what areas remain uniquely human. To stay competitive, physicians should strengthen the expertise that AI cannot easily replace, while also leveraging AI to expand their competence.” (SME 3, Early)  “There are already cases of AI misuse—overreliance, really. The FDA recently warned against using AI not designed for triage as a triage tool.” (SME 4, Early)  “What really matters is having strong domain knowledge to judge whether AI’s results make sense. Doctors don’t need to build AI—they need to interpret and evaluate its output using their expertise.” (SME 5, Mid)  “In prosthodontics... if those basics are neglected, they won’t be able to tell whether an AI-generated design aligns with fundamental dental principles.” (SME 7, Early)  “In the end, developing an algorithm is really all about the data... the annotation process—how you label and define the correct answers—needs to be done well... With explainable AI (XAI), you can look at the output and think, ‘Okay, this model made this judgment based on that data—so maybe if we include more of this kind of data, the performance will improve.’ That kind of understanding is really valuable.” (SME 13, Early) | “AI solutions will continue to diversify, and each one will have its own type of data bias. Physicians will face situations where they must decide which solution best fits a given patient.” (SME 1, Early)  “AI is basically an IT solution. They need to distinguish between the AI platform itself and the software solutions developed on that platform. For example, Watson is IBM’s AI platform, and Watson for Oncology is a solution built on it. AI solutions isn’t much different from Microsoft Word or PowerPoint, except that it provides a certain degree of medical expertise—partly, not fully. That’s the right way to see it.” (SME 3, Early)  “To diagnose and treat patients properly, you ultimately need to understand which tool is the best one.” (SME 4, Early)  “You know how we usually ask around—‘maybe that person knows?’ or ‘should I ask someone else?’ With AI, instead of asking five people, you might only need to ask two or three. It makes consultations way more efficient. You can quickly figure out who to refer to, so the whole referral process becomes smoother. What used to take a full round of visits or a week to follow up now happens much faster—the workflow just flows better.” (SME 5, Mid)  “Honestly, communication is the hardest part. No matter how fancy the software is, it won’t work if the users can’t communicate effectively with those who build or maintain it. For example, when schedules or workflows need to be adjusted, algorithms might automatically allocate resources—but clinicians often want flexibility that the system doesn’t yet support. That’s where frustration arises.” (SME 8, Mid)  “Clinicians and developers speak completely different languages… Clinicians talk about clinical meaning, while developers talk about accuracy scores… education that bridges that gap…” (SME 10, Mid)  “We need to educate physicians on what to do when AI fails. In digital dentistry, for instance, if a problem can’t be solved with advanced technology, you must know how to revert to earlier methods—even manual ones. Understanding that flow helps you judge whether a limitation comes from lack of knowledge or from the tool itself.” (SME 12, Mid) | “Ultimately, AI can function as a tool to optimize resource utilization across hospitals and healthcare systems, rather than focusing solely on the individual patient level. Future healthcare leaders should be trained to understand resource management from this systems-oriented perspective.” (SME 1, Early)  “AI leadership requires the capacity to plan beyond technology—to design interconnected infrastructures, workforce systems, and policies that support these changes. Education for AI leaders must therefore cultivate systemic thinking, policy implementation skills, and integrated management of resources and human capital.” (SME 3, Early)  “An AI leader isn’t just someone who holds a position — it’s someone with the vision... to set the direction... [and] draw the big picture...” (SME 5, Mid)  “In medical systems, we need to clearly define who holds responsibility, risk, and transparency. What are the roles of developers, planners, and users? How should accountability be shared among them? These questions must guide national-level AI governance frameworks and should be explicitly addressed in AI leadership education.” (SME 6, Late)  “The key question isn’t whether a technology can be realized, but whether society is ready... AI leadership education must address... social acceptance, ethical judgment, and systemic policy awareness.” (SME 8, Mid)  “When applying AI in clinical environments, customizing the setup to each hospital’s context is crucial… involving IT [information technology] teams, EMR [electronic medical record] administrators, and support departments… and designing multidisciplinary governance structures.” (SME 9, Late)  “Regulatory expectations are extremely high... There’s a huge gap between research-grade models and commercially approved ones... Developer education should... train students to understand international regulatory systems.” (SME 11, Late) |
| *Note:* Across the interview period, the emphasis of expert concerns shifted—foundational issues were most prominent in earlier interviews, and system-level considerations were elaborated in greater specificity later—yet all three emphases were present throughout: early-phase experts (SME 1, 3) already articulated governance-level concerns, and foundational concerns persisted into mid-phase interviews (SME 5). Later interviews introduced no new role categories; this temporal consistency supports the durability of the role-differentiated framework.  SME numbers indicate experts voicing each emphasis (interview phase in Table 1); **bold** = dominant, defining emphasis in that expert’s account; non-bold = also raised.  **Interview and member-checking timeline of the 13 subject matter experts (October 2022–October 2025)** | | |
